# A case of *Cardiobacterium valvarum* endocarditis with cerebral hemorrhage after MVR, TVP and vegetation removal operation

**DOI:** 10.1186/s12941-018-0263-z

**Published:** 2018-03-22

**Authors:** Lijia Ni, Xiaoying Xie, Nengyong Ouyang, Baiji Chen, Dongye Wang, Xiaoqiang Liu, Xiquan Wu, Jiajian Guo, Hongyu Li, Yandan Yao, Songyin Huang

**Affiliations:** 1 Department of Clinical Laboratory, Sun Yat-Sen Memorial Hospital, Sun Yat-Sen University, Guangzhou, 510120 China; 2 Guangdong Provincial Key Laboratory of Malignant Tumor Epigenetics and Gene Regulation, Sun Yat-Sen Memorial Hospital, Sun Yat-Sen University, Guangzhou, 510120 China

**Keywords:** *Cardiobacterium valvarum*, Endocarditis, Cerebral hemorrhage

## Abstract

**Background:**

*Cardiobacterium* is a fastidious Gram-negative bacillus, and is a rare human pathogen in clinical settings. Herein, we describe a case of *Cardiobacterium valvarum* (*C. valvarum*) endocarditis with a rare complication of cerebral hemorrhage after mitral valve replacement (MVR), tricuspid valve prosthesis (TVP) and vegetation removal operation.

**Case presentation:**

A 41-year-old woman who had a history of gingivitis developed into infective endocarditis due to the infection of *C. valvarum*. Then, she was hospitalized to receive MVR, TVP and vegetation removal operation. The indicators of patient tended to be normal until the abrupt cerebral hemorrhage occurred on day 15 after operation. This is the first well-described case of *C. valvarum* infection in China, and the first report of *C. valvarum* endocarditis with cerebral hemorrhage after MVR, TVP and vegetation removal operation worldwide.

**Conclusions:**

We reported the first case of *C. valvarum* infection in China clinically, with a rare complication of cerebral hemorrhage after MVR, TVP and vegetation removal operation.

## Background

The genus *Cardiobacterium* encompasses two species, *Cardiobacterium valvarum* (*C. valvarum*) and *Cardiobacterium hominis* (*C. hominis*), with the latter one having higher infection prevalence. *C. valvarum* is a fastidious Gram-negative bacillus, and is a rare human pathogen in clinical settings. Only 13 cases were reported to date since 2004 cross the whole world [1–13]. Therefore, the clinical characteristics of *C. valvarum* infection are not yet fully understood. Identification of *C. valvarum* is usually difficult because of its phenotypic characteristics, and its identification may require the use of reference laboratories with molecular identification techniques. In this work, we present a case of infective endocarditis in a 41-year-old woman with mitral and tricuspid valve insufficiency. *C. valvarum* was isolated from the blood before MVR, TVP and vegetation removal operation. This is the first well-described case of *C. valvarum* infection in China, and the first report of *C. valvarum* endocarditis with cerebral hemorrhage after MVR, TVP and vegetation removal operation worldwide.

## Case presentation

A 41-year-old woman was hospitalized because of 1 week history of worsening tachypnea and post-exercising chest congestion, and 2 days of severe pain in back. The patient had a medical history of hyperthyroidism diagnosed more than 10 years ago, as well as a medical history of right breast fibroma diagnosed 3 years ago, for which she received a minimally invasive surgical operation, and her post-operative condition had been stable. Two weeks ago, she visited a dentist for gingivitis, which was successfully controlled with antibiotics and drainage therapies.

Her primary diagnosis of admission to hospital is valvular disease. And at initial presentation, the patient had a body temperature of 36.6 °C, blood pressure of 132/82 mmHg, and pulse of 75 beats per min. Upon physical examination, diastolic murmurs were heard along the left sternal border, loudest in the third or fourth left intercostal space. Laboratory tests revealed a normocytic normochromic anemia, with haemoglobin at 95 g/L. Serum rheumatoid factor (RF) was elevated (207.0 IU/mL) when admission. Low serum albumin level (29.1–32.9 g/L), low serum iron (5.4–6.1 µmol/L) and low serum cholinesterase level (3855–4029 U/L) were detected throughout the hospitalization.

**Fig. 1:**
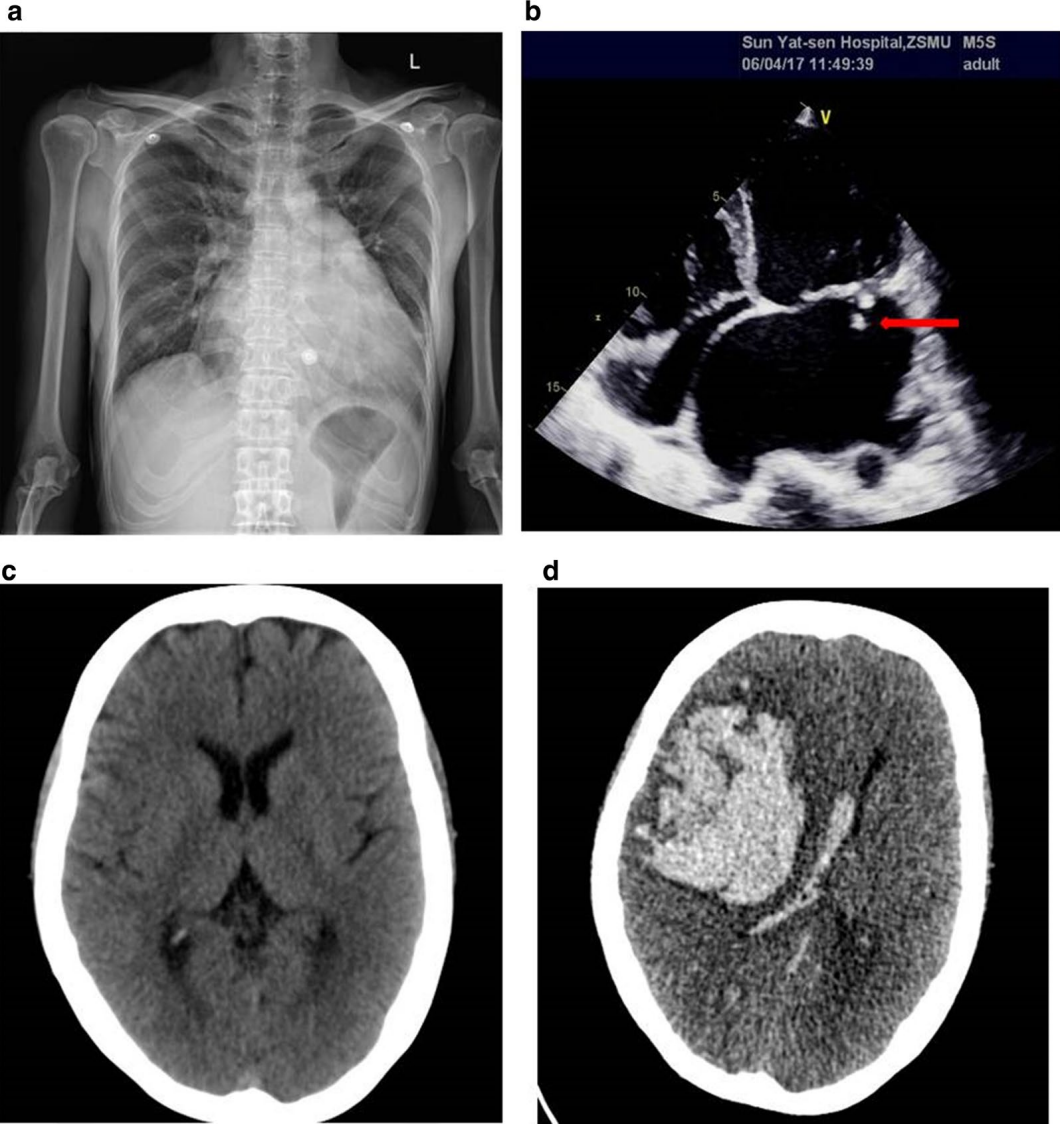
Image examination of the patient. **a** The chest X-ray plain film showed cardiac image enlargement in left atrium, right ventricle and left ventricle, together with bilateral pulmonary congestion. **b** Echocardiography examination showed mitral valve prolapse and multiple vegetations (the largest one was about 14 mm, arrow). **c** Computerized tomography scan of the brain showed mild ischemia at bilateral basal ganglia. **d** Computerized tomography scan of the brain showed massive intracerebral hemorrhage in the right frontal–temporal lobe and lateral ventricle

Blood cultures were sampled after admission (aerobic and anaerobic bottles; two sets of cultures). In the meantime, ceftizoxime sodium of 3 g twice per day for 7 days as empiric antibiotic therapy was administrated to the patient until the operation. The two aerobic blood cultures became positive for Gram-negative bacilli after an incubation period of 74–80 h in the Biomerieux BacT/Alert 3D blood culturing system. The chest X-ray plain film showed cardiac image enlargement, obviously in left atrium, right ventricle and left ventricle, together with bilateral pulmonary congestion (Fig. 1a). Echocardiography examination showed mitral valve prolapse and multiple vegetations, and the largest one was about 14 mm. In addition, severe mitral regurgitation, and tricuspid valve prolapse, together with moderate regurgitation and pulmonary hypertension were also observed, indicating infectious endocarditis (Fig. 1b). At the same period, the computerized tomography (CT) scan of the brain was performed and showed mild ischemia at bilateral basal ganglia (Fig. 1c).

With the results of blood cultures and image examinations, the diagnosis of “infectious endocarditis, mitral insufficiency and tricuspid insufficiency” was made. And then, the patient received MVR, TVP and vegetation removal operation under general anesthesia on day 7 of admission. In order to control postoperative infection, meropenem of 1 g every 8 h were administrated to the patient for 10 days according to the susceptibility testing, and the increasing CRP level and elevated total white blood cell began to decrease to normal after 5 days of antibiotic treatments. And then the antibiotic administration was changed to ceftizoxime sodium of 3 g twice per day for 5 days until the abrupt cerebral hemorrhage occurred on the 15th day after operation. The coagulation test and the platelet aggregation function test were normal on admission. Warfarin of 3 mg per day was taken for anticoagulation treatment since day 2 after operation, and the international normalized ratios (INRs) were 1.6–2.76. Notably, the platelet showed increase since day 7 (405–622 × 10^9^/L) after operation together with the high platelet aggregation function (52.4%). Infectious markers and coagulative function indicators before and after the operation were listed in Table 1. Blood cultures kept negative since the antibiotic treatments. After aforementioned treatment, the patient did not have a fever, cough, dyspnea or chest pain, under a stable condition, except complaining about slight dizziness and headache infrequently.

**Table 1:** Infectious markers and coagulative function indicators before and after the operation

	Before operation	After operation	1 day after operation	2 day after operation^C^	5 day after operation^D^	10 day after operation	11 day after operation	13 day after operation	14 day after operation	15 day after operation^E^
CRP (mg/L)	75.0		49.2	125.9	119.1	36.4	42.2	34.3	18.6	23.4
WBC (× 10^9^/L)	8.70	21.87	17.73	23.64	13.48	10.19	8.44	8.41	7.88	17.1
N (%)	71.2	90.0	93.7	86.0	79.9	61.9	57.2	57.8	52.7	76.9
Hb (g/L)	95	92	110	112	121	112	112	114	111	104
HCT	0.299	0.279	0.327	0.354	0.378	0.343	0.346	0.352	0.340	0.314
PLT (× 10^9^/L)	346	212	234	277	325	555	584	611	599	562
PAGT (%)^A^	45.31						52.37			
PT (s)	14.0	15.3	12.9	13.4	22.5	31.2	31.6	19.0	18.5	23.8
PTA (%)^B^	57.0	48.8	68.7	63.0	28.7	19.2	18.9	35.4	36.8	26.8
INR	1.20	1.31	1.10	1.15	1.95	2.72	2.76	1.64	1.60	2.06
Fbg (g/L)	3.47	2.38	3.03							
APTT (s)	30.9	36.0	30.9							
TT (s)	18.5	19.9	19.7							
DD (mg/L)	1.93	4.14	2.44							

PS:CRP normal range is less than 5 mg/L; WBC normal range is (3.50–9.50) × 10^9^/L; N normal range is (40.0–60.0) %; Hb normal range is (115–150) g/L; HCT normal range is 0.400–0.500; PLT normal range is (125–350) × 10^9^/L; PAGT normal range is (37.30–47.50) %; PT normal range is (10.7–14.4) s; PTA normal range is (70.0–130.0) %; INR normal range is 0.82–1.15; Fbg normal range is (2.00–4.00) g/L; APTT normal range is (23.5–35.0) s; TT normal range is (14.0–21.0) s, DD normal range is less than 0.55 mg/L

^A^PAGT is short for platelet aggregation function, and platelet aggregation induced by ADP

^B^PTA is short for prothrombin activity

^C^Warfarin of 3 mg per day was taken for anticoagulation treatment since day 2 after operation

^D^Warfarin was effected since day 5 after operation

^E^Cerebral hemorrhage occurred on the 15th day after operation, 13 days after warfarin was taken

On the early morning of day 23 of admission, 15 days after operation, a sudden loss of consciousness together with bilateral mydriasis occurred. At that time, the patient had a high blood pressure of 180/90 mmHg, heart rate of 69 beats per min, and breathing rate of 15 times per minute. Antihypertensive therapy, temporary pacemaker and tracheotomy auxiliary ventilation were used for symptomatic supportive therapy, and the patient’s condition tended to be stable. An urgent computerized tomography (CT) scan of the brain showed large high intensity areas at right frontal lobe, parietal lobe and temporal lobe that indicated spontaneous cerebral hemorrhage and broken into bilateral ventricles combined with hernia, together with subarachnoid hemorrhage (Fig. 1d). An urgent large craniectomy hematoma operation was performed on the day, and then the patient was transferred to the intensive care unit for observation. During the surgery, a neoplasm (1.1 cm × 1.0 cm × 0.7 cm) was found in the right temporal lobe, and pathologically diagnosed as an intravascular thrombus. After the urgent large craniectomy hematoma operation, the patient fell into a stupor. Necessary nursing and treatment were kept, but the patient remained in a coma. During the long-term mechanical ventilation of intensive care unit, the patient obtained the lung infection of *Acinetobacter baumannii*. After the infection was controlled, the patient was transferred to the neurology department for continued nutrition support therapy. Eventually, the patient was discharged after 2 months of treatment.

## Microbiology investigations

**Fig. 2:**
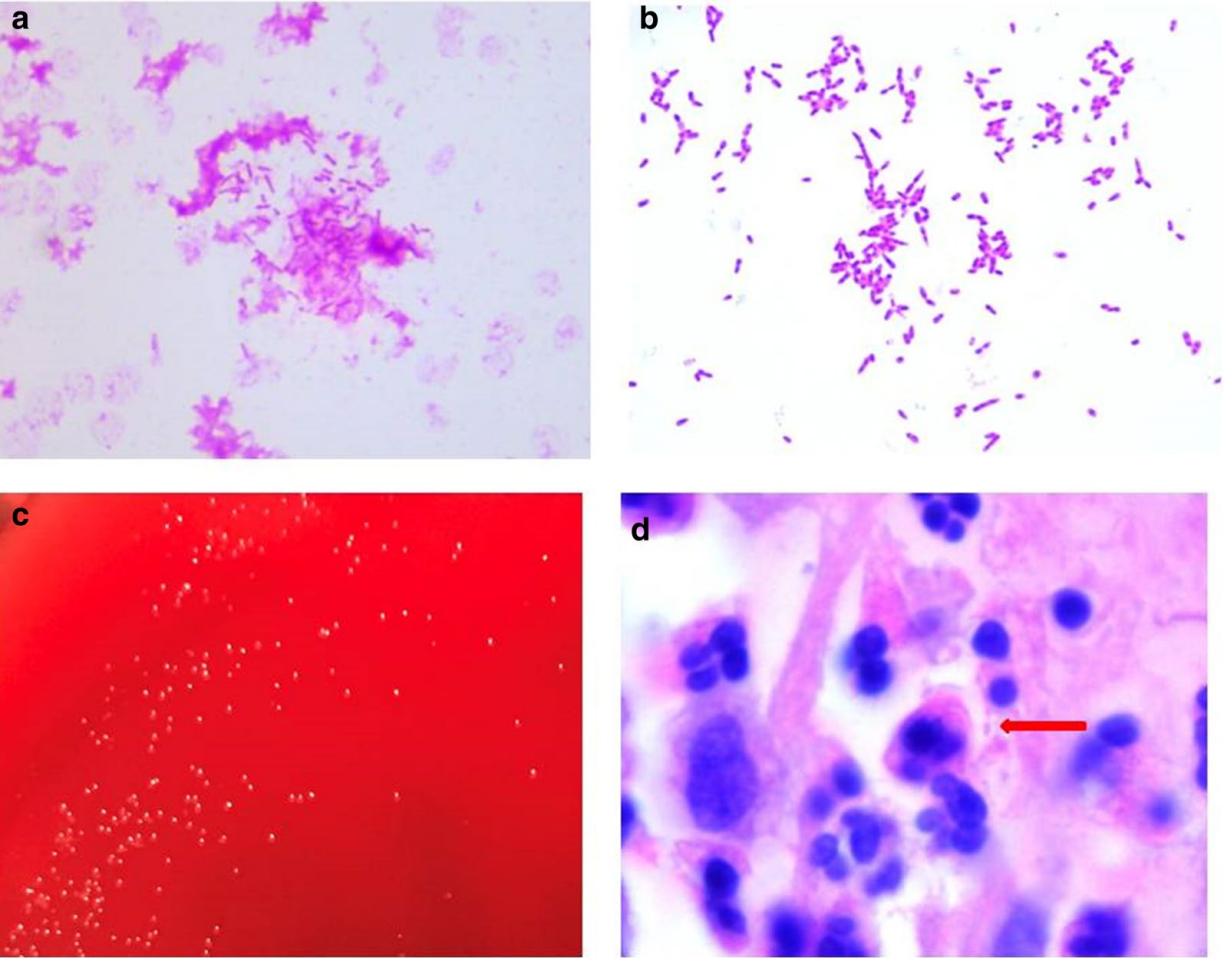
stain morphology (magnification, ×1000). **a** Gram stains from blood culture; **b** Gram stains from agar with sheep blood. **c** Colony morphology of *C. valvarum* on blood agar after 4 days of growth at 37 °C with 5% CO_2_; **d** small bacillus in the valvular vegetations slides were found by microscopy (arrow)

Bacterial strains were isolated from blood samples using the Bact/Alert 3D system (BioMerieux, Marcy l’Etoile, France). In this case, the strains yielded growth in the two aerobic blood cultures became positive after 3 days of incubation. Microscopy of the blood culture fluid identified small, rounded at both ends, Gram-negative, pleomorphic bacillus, often arranged in clusters (Fig. 2a, b). When it grows on 5% sheep blood agar as well as chocolate agar (BioMerieux, Marcy l’ Etoile, France) and incubating in ambient air supplemented with 5% CO_2_, the strains grew with gray white mycoderm after 24 h incubation and with glistening colonies of 0.5 mm after 4 days incubation. Colonies were similar, being small, round, opaque, smooth, grey and weakly alpha-hemolytic on blood agar plates (Fig. 2c). In order to clarify the source of pathogens, the oral cavity swab and valvular vegetations were cultured, while no target pathogens were found. However, some small, Gram-negative bacillus in the valvular vegetations slides were found by microscopy (Fig. 2d).

Both conventional phenotype-based identification by VITEK^®^2 (BioMerieux, Marcy l’Etoile, France) and Matrix-assisted laser desorption/ionization time-of-flight mass spectrometry (MALDI-TOF) by VITEK^®^MS (BioMerieux, Marcy l’ Etoile, France) failed to identify the bacteria isolated from the blood. 16S rRNA gene sequencing analysis was performed as described previously using two universal bacterial primers, 1492R (5′-GGTTACCTTGT TACGACTT-3′) and Eubac27F (5′-AGAGTTTGATCCTGGCTCAG-3′) [14]. The amplified DNA fragments and DNA sequencing were performed according to the manufacture’s instruction (BIO-RAD, California, USA). After analyzing the sequence using the BLAST at GenBank, the two isolates were confirmed to have 97.19% homology with the published sequence of *C. valvarum* strain (GenBank No. NR_028847.1), genetically identified as being *C. valvarum.*

Antibiotic susceptibility testing was performed with E test (BOI-KONT, Zhejiang, China; AutoBio, Zhengzhou, China) on cation-adjusted Mueller–Hinton broth (CAMHB) with lysed horse blood (LHB) (2.5 to 5% v/v). The minimal inhibitory concentrations (MICs) were presented in Table 2 and interpreted according to CLSI M45-A. The *C. valvarum* strain showed high susceptibility to the listed antibiotics.

**Table 2:** The MICs of *C. valvarum* susceptibility results which isolated from the blood

Antibiotics	MIC (µg/mL)	Interpretation
Penicillin G	0.064	S
Ampicillin	0.016	S
Amoxicillin–clavulanic acid	0.016	S
Cefotaxime	1	S
Meropenem	0.002	S
Imipenem	0.006	S
Levofloxacin	0.024	S
Ciprofloxacin	0.016	S
Tetracycline	0.094	S
Chloramphenicol	0.25	S
Rifampin	0.064	S
Trimethoprim–sulfamethoxazole	0.032	S

## Discussion

The genus *Cardiobacterium* is fastidious, Gram-negative bacillus, normal upper respiratory flora in humans, which encompasses two species: *Cardiobacterium valvarum* (*C. valvarum*) and *Cardiobacterium hominis* (*C. hominis*) [15]. The phenotypic profiles of the two species are very similar, so it is difficult to identify by conventional methods. MALDI-TOF [16, 17] and 16S rRNA gene sequencing [18] have emerged as more accurate and reliable methods for identifying these rare microorganisms. In contrast to *C. hominis,* rare case reports and information about *C. valvarum* were published since its first identification in 2004 [7], so its clinical implication is not as clear as the former. All the published *C. valvarum* strains were identified by 16S rRNA gene sequencing, including this case, while MALDI-TOF can only provide a presumptive but not conclusive identification [2–4], which may be attributed to the imperfect bacterial library.

As in the previous cases published, our case developed infectious endocarditis as well, and large, devastating valvular vegetations formed, which had been reported as the characteristic change in HACEK bacteria [1, 2, 4, 19]. Additionally, this case visited the dentist due to gingivitis 2 weeks before admission, which is similar to the previous cases showing recent dental work or poor dentition were commonly associated risk factors [3–5, 7, 11]. Initial presentation of previous patients was often non-specific and without fever. Blood cultures were mostly negative at 48 h with an incubation period ranging from 3 to 5 days, which were 74–80 h in this case. Notably, an abrupt cerebral hemorrhage with herniation occurred in this case, and an intravascular thrombus was found in the right temporal lobe, never reported before. Other neurological complications were common in the previous cases including cerebral infarction [1, 4, 6], subarachnoid hemorrhage [4, 7, 9], cerebral vasculitis [1] and cerebral embolization [2]. Neurological sequelae may be another clinical feature of *C. valvarum* bacteria.

In order to clarify the source of pathogens, the oral cavity swab and valvular vegetations were cultured, while no positive findings. This may be the reason that conventional bacterial cultures are not suitable for the growth of target bacteria, as well as the experience of isolating this pathogen is lacking. However, we found some small, Gram-negative bacillus in the valvular vegetations slides by microscopy, which further indicated that *C. valvarum* could be the pathogen of the endocarditis in this case. *C. valvarum* grows slowly and visible small colonies (hardly reaching 1 mm) appear only after incubation for 3–4 days. To understand the true clinical picture of *C. valvarum* infection, a molecular diagnostic method is indispensable. In this case, analysis using 16S rRNA gene sequences showed the strains isolated from blood had 97.19% homology with the published sequence of *C. valvarum* strain, meanwhile, 92.04% homology with *C. hominis* (GenBank No. NR_025934.1), finally identified as the former. We speculate there may be mutation of the strain in our case, so the homology was not as high as the previous cases, major of which were higher than 99% [1–4, 6, 7, 18, 20].

*Cardiobacterium valvarum* has been reported to be susceptible to many antibiotics. In our case, antimicrobial susceptibility testing using the E-test showed that the isolate was sensitive to penicillins and β-lactam/β-lactamase inhibitor combinations, cephalosporins, carbapenems, quinolones, tetracyclines, phenicols, ansamycins and folate pathway inhibitors. Most of the previous cases were treated with β-lactams and their prognoses were favorable. Our patient received third-generation cephalosporins as empiric antibiotic therapy for 1 week and carbapenems as well as third-generation cephalosporins according to the susceptibility testing for approximately 2 weeks and the infection was controlled.

Oral anticoagulation is essential to prevent thromboembolic events (TEs) especially for the patients with mechanical heart valves. On the other hand, anticoagulation therapy is also associated with an increased risk of bleeding complications. Studies showed that the patients treated by warfarin would stay in the safest condition with INRs between 2.0 and 2.5 [21, 22], the morbidity of bleeding was significantly increased with an INR > 2.5 [14], and the incidence of TEs was significantly higher with an INR < 2.0 [22]. In China, Zhang et al. [23]. reported that the incidence of total complications was the lowest with INRs of 1.8–2.3 in the mitral valve replacement and double valve replacement patients. In our case, the patient had cerebral hemorrhage in the right frontal–temporal, parietal, and an intravascular thrombus in the right temporal lobe during the oral anticoagulation treatments. The INRs throughout anticoagulation therapy were 1.60–2.76, a respectively safe range. With no changes in warfarin’s anticoagulant therapy, the INRs went down from more than 2.5 to less than 1.8 for 2 days, before the thrombus and cereal bleeding occurred. In the meantime, platelet showed sustained increase throughout anticoagulation therapy together with the high platelet aggregation function. So we suspected that the patient stayed in a hypercoagulative state before cereal bleeding event, which lead to the thrombus. As the secondary event, the bleeding occurred due to the extreme consumption of coagulation factors and platelets after thrombus. What’s more, the patient had an extremely high systolic pressure of 180 mmHg at the moment of sudden cerebral hemorrhage. Above all, it is important to control the INR within the appropriate range carefully, pay close attention to the blood pressure and coagulation function state after mechanical valve replacement.

## Conclusions

In summary, our case of *C. valvarum* infection is the first case in China to be described in detail, as well as the first case accompanying spontaneous cerebral hemorrhage. This case also demonstrates that, the history of dental diseases, common features, a large vegetation burden, neurological sequelae, and slow growth pathogen in blood culture (always > 3 days) are the main clinical indications of *C. valvarum* infection.
